# Diffusion Kurtosis Imaging for Detection of Early Brain Changes in Parkinson's Disease

**DOI:** 10.3389/fneur.2019.01285

**Published:** 2019-12-10

**Authors:** Jitian Guan, Xilun Ma, Yiqun Geng, Dan Qi, Yuanyu Shen, Zhiwei Shen, Yanzi Chen, Erxi Wu, Renhua Wu

**Affiliations:** ^1^Department of Radiology, The Second Affiliated Hospital of Shantou University Medical College, Shantou, China; ^2^Department of Neurosurgery, Baylor Scott & White Health, Temple, TX, United States; ^3^Neuroscience Institute, Baylor Scott & White Health, Temple, TX, United States; ^4^Department of Radiology, The First Affiliated Hospital of Shantou University Medical College, Shantou, China; ^5^Laboratory of Molecular Pathology, Shantou University Medical College, Shantou, China; ^6^Department of Surgery, Texas A&M University Health Science Center College of Medicine, Temple, TX, United States; ^7^Department of Pharmaceutical Sciences, College of Pharmacy, Texas A&M University Health Science Center, College Station, TX, United States; ^8^Dell Medical School, LIVESTRONG Cancer Institute, The University of Texas at Austin, Austin, TX, United States

**Keywords:** diffusion kurtosis imaging, magnetic resonance imaging, Parkinson's disease, red nucleus, substantia nigra

## Abstract

We aimed to evaluate microscale changes in the bilateral red nucleus and substantia nigra of patients with Parkinson's disease (PD) using diffusion kurtosis imaging (DKI). Twenty-six patients with PD [mean age, 62.5 ± 8.7 years; Hoehn-Yahr stage, 0–4.0; Unified Parkinson's Disease Rating Scale (UPDRS) scores, 8–43] and 15 healthy controls (mean age, 59.5 ± 9.4 years) underwent DKI of the substantia nigra and red nucleus. Imaging was performed using a General Electric (GE) Signa 3.0-T MRI system. Patients with PD were divided into two groups consisting of 12 patients with UPDRS scores ≥ 30 and 14 patients with UPDRS scores < 30. All DKI data processing operations were performed with commercial workstations (GE, ADW 4.6) using Functool software to generate color-coded and parametric maps of mean kurtosis (MK), fractional anisotropy (FA), and mean diffusivity (MD). MK values in the bilateral substantia nigra were significantly lower in patients with early- and advanced-stage PD than in controls. Moreover, MK values in the left substantia nigra were significantly lower in patients with advanced-stage PD than in those with early-stage PD. Patients with advanced-stage PD also exhibited significant decreases in MK values in the bilateral red nucleus relative to controls. No significant differences in FA or MD values were observed between the PD and control groups. There were no significant correlations between MK, FA, or MD values and UPDRS scores. Our findings suggest that decreased MK values in the substantia nigra may aid in determining the severity of PD and help provide early diagnoses.

## Introduction

Parkinson's disease (PD) is a progressive neurodegenerative disease caused by the degeneration of nigrostriatal dopaminergic neurons. The main pathological changes associated with PD include the loss of dopaminergic neurons in the substantia nigra/striatum and the presence of Lewy neuritis, which result in movement disorders. However, the initial diagnosis of PD is based on cardinal signs such as bradykinesia, resting tremor, postural instability, gait disorder, and muscular rigidity, all of which become evident only after an 80% loss of dopaminergic neurons in the striatum (1, 2). In the recent years, many laboratory tests (e.g., cerebrospinal fluid analysis, blood tests) have been used to provide excellent support for the early prediction of PD. However, use of these biomarkers alone is not sufficient for diagnosis, as PD is not a disease with a single characteristic; specific biomarkers for motor and non-motor dysfunction would lead to more precise and individualized treatment for patients with PD. Combining findings from different fields may assist in identifying them. More sensitive diagnostic methods are therefore required to ensure timely and appropriate treatment.

Diffusion tensor imaging (DTI), a non-invasive MRI technique based on diffusion-weight imaging (DWI), has been used for *in vivo* assessments of white matter integrity. To date, both DTI and DWI have been applied for the diagnosis of various diseases (3, 4). Previous studies have allowed for the evaluation of conventional diffusion tensor metrics such as mean diffusivity (MD) and fractional anisotropy (FA). Several studies using DTI revealed microstructural alterations in the white matter in PD patients with motor impairment (5–7). DTI is also used to examine gray matter areas. Several studies measuring DTI parameters such as FA have shown that FA in the substantia nigra is reduced in patients with PD compared to healthy controls, which indicates that FA may be a diagnostic biomarker of PD (8–10). However, the results using FA are not always consistent. Authors of another study observed that the FA in the substantia nigra of PD patients did not differ significantly from that of controls (11). While measuring MD targeting the substantia nigra, one study showed that increased MD in this region helped distinguish patients with PD from those with multiple system atrophy (12). In contrast, other studies found that nigral MD changes had no significant effect on the disease (13, 14). Diffusion kurtosis imaging (DKI) can provide other metrics related to non-Gaussian water diffusion such as mean kurtosis (MK) (15). In general, MK is related to the complexity of the microstructure. A previous study demonstrated that MK was significantly increased in the substantia nigra of PD patients; MK in the substantia nigra was thought to be a sensitive metric for early diagnosis of PD (16).

In the present study, we utilized DKI to evaluate microscale changes in the substantia nigra and red nucleus of patients with PD and to determine how these regions are altered at different stages of PD.

## Results

### General Characteristics of Study Participants

There were no significant differences in age (*F* = 0.224, *p* = 0.303) or gender (*X*^2^ = 0.383, *p* = 0.536) between the PD and control (CTL) groups. Furthermore, there were no significant differences in age (*F* = 0.949, *p* = 0.625) or gender (*X*^2^ = 2.872, *p* = 0.238) among the early-stage PD, advanced-stage PD, and CTL groups.

### Comparison of MK, FA, and MD Values in the Substantia Nigra and Red Nucleus

**Figure 1:**
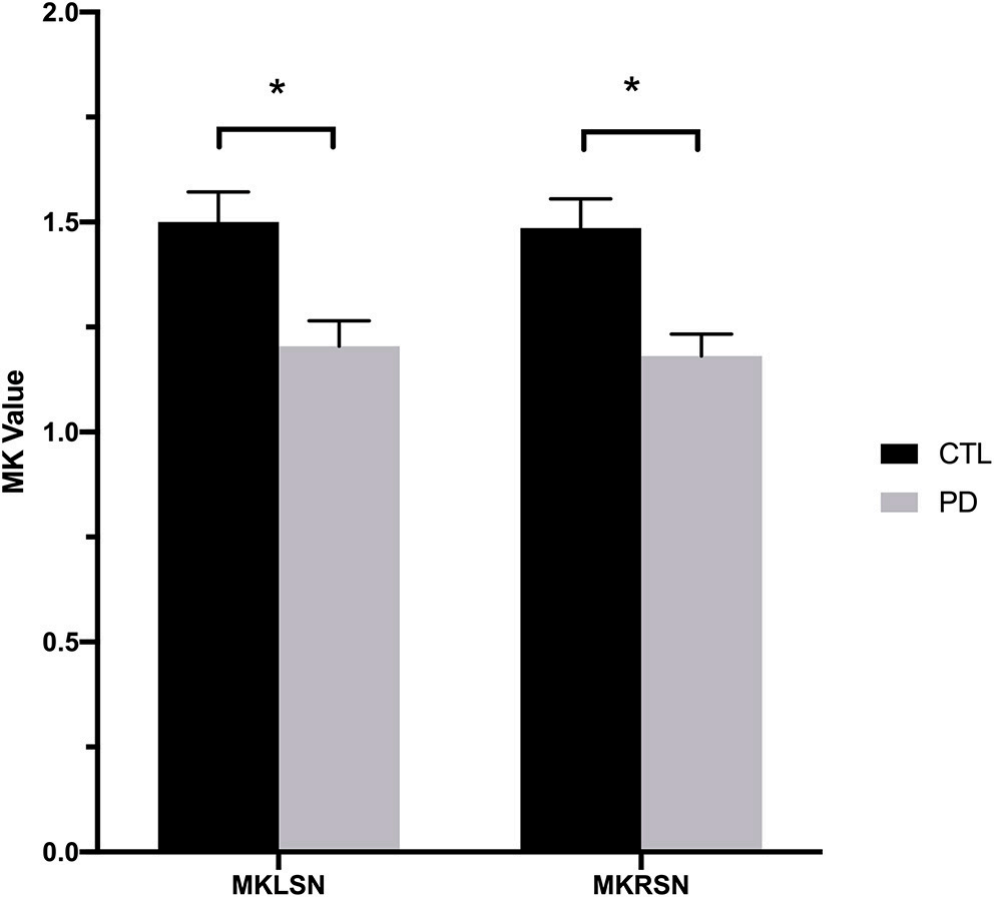
Mean kurtosis values for the bilateral substantia nigra in the Parkinson's disease and control groups. CTL, control; MKLSN, mean kurtosis of the left substantia nigra; MKRSN, mean kurtosis of the right substantia nigra; PD, Parkinson's disease. *p < 0.05.

**Figure 2:**
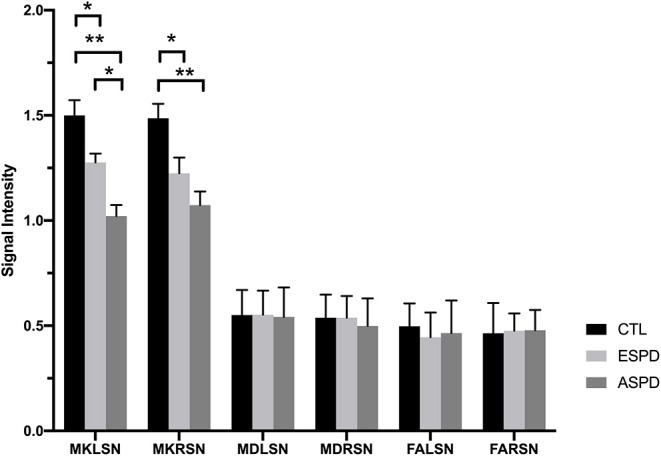
Diffusion kurtosis imaging values in the substantia nigra for the control, early-stage Parkinson's disease, and advanced-stage Parkinson's disease groups. ASPD, advanced-stage Parkinson's disease; CTL, control; ESPD, early-stage Parkinson's disease; FALSN, fractional anisotropy of the left substantia nigra; FARSN, fractional anisotropy of the right substantia nigra; MDLSN, mean diffusivity of the left substantia nigra; MDRSN, mean diffusivity of the right substantia nigra; MKLSN, mean kurtosis of the left substantia nigra; MKRSN, mean kurtosis of the right substantia nigra. **p < 0.01, ASPD vs. CTL; *p < 0.05, early-stage PD vs. CTL or advanced-stage PD vs. CTL or advanced-stage PD vs. early-stage PD.

**Figure 3:**
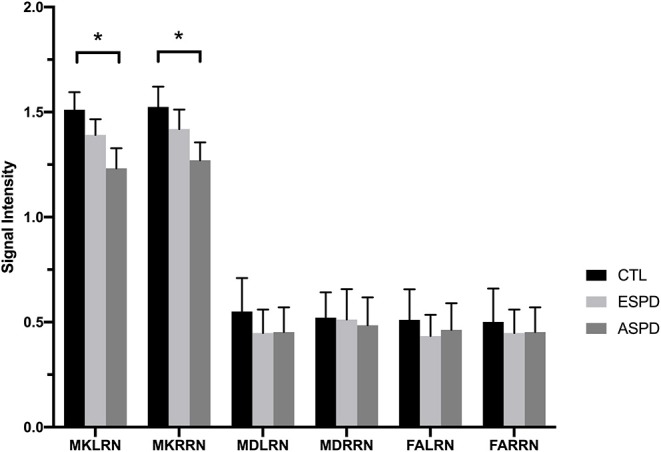
Diffusion kurtosis imaging values in the red nucleus for the control, early-stage Parkinson's disease, and advanced-stage Parkinson's disease groups. ASPD, advanced-stage Parkinson's disease; CTL, control; ESPD, early-stage Parkinson's disease; MDLRN, mean diffusivity of the left red nucleus; FALRN, fractional anisotropy of the left red nucleus; FARRN, fractional anisotropy of the right red nucleus; MDRRN, mean diffusivity of the right red nucleus; MKLRN, mean kurtosis of the left red nucleus; MKRRN, mean kurtosis of the right red nucleus. *p < 0.05, ESPD vs. CTL or ASPD vs. CTL or ASPD vs. ESPD.

MK values in the substantia nigra were significantly lower in patients with early- and advanced-stage PD than in controls (Figure 1, Table 1). In addition, MK values in the left substantia nigra were significantly lower in patients with advanced-stage PD than in patients with early-stage PD (Figure 2, Table 2). MK values in the bilateral red nucleus were significantly lower in patients with advanced-stage PD than in controls (Figure 3, Table 2). No significant differences in FA or MD values were observed between the PD and CTL groups (Figures 2, 3, Table 2).

**Table 1:** Diffusion kurtosis imaging values in the bilateral red nucleus and substantia nigra in the Parkinson's disease and control groups.

**DKI values**	**PD (*N* = 26)**	**CTL (*N* = 15)**	***p* value**
	**Mean ± SD**	**Mean ± SD**	
**Left SN**			
MK	1.204 ± 0.061	1.500 ± 0.072	0.022^*^
MD	0.547 ± 0.125	0.551 ± 0.119	0.934
FA	0.454 ± 0.134	0.497 ± 0.109	0.296
**Right SN**			
MK	1.181 ± 0.052	1.486 ± 0.069	0.023^*^
MD	0.520 ± 0.116	0.538 ± 0.110	0.617
FA	0.477 ± 0.087	0.464 ± 0.144	0.728
**Left RN**			
MK	1.336 ± 0.247	1.451 ± 0.184	0.127
MD	0.517 ± 0.114	0.551 ± 0.159	0.440
FA	0.447 ± 0.112	0.511 ± 0.145	0.125
**Right RN**			
MK	1.407 ± 0.251	1.525 ± 0.188	0.119
MD	0.500 ± 0.137	0.521 ± 0.121	0.622
FA	0.450 ± 0.112	0.501 ± 0.159	0.241

*CTL, control; DKI, diffusion kurtosis imaging; FA, fractional anisotropy; MD, mean diffusivity; MK, mean kurtosis; PD, Parkinson's disease; RN, red nuclei; SN, substantia nigra; SD, standard deviation*.

* *p < 0.05, PD vs. CTL*.

**Table 2:** Diffusion kurtosis imaging values in the bilateral red nucleus and substantia nigra in the control, early-stage Parkinson's disease, and advanced-stage Parkinson's disease groups.

**DKI values**	**CTL (*N* = 15)**	**Early-stage PD** **(*N* = 14)**	**Advanced-stage** **PD (*N* = 12)**
	**Mean ± SD**	**Mean ± SD**	**Mean ± SD**
**Left SN**			
MK	1.500 ± 0.072	1.276 ± 0.042^*^	1.021 ± 0.053^**^
MD	0.551 ± 0.119	0.552 ± 0.115	0.542 ± 0.140
FA	0.497 ± 0.109	0.445 ± 0.118	0.465 ± 0.155
**Right SN**			
MK	1.486 ± 0.069	1.225 ± 0.074^*^	1.073 ± 0.065^**^
MD	0.538 ± 0.110	0.538 ± 0.103	0.499 ± 0.131
FA	0.464 ± 0.144	0.476 ± 0.082	0.478 ± 0.097
**Left RN**			
MK	1.511 ± 0.084	1.391 ± 0.075	1.232 ± 0.096^*^
MD	0.551 ± 0.159	0.448 ± 0.112	0.453 ± 0.117
FA	0.511 ± 0.145	0.434 ± 0.101	0.463 ± 0.127
**Right RN**			
MK	1.525 ± 0.096	1.419 ± 0.093	1.271 ± 0.085^*^
MD	0.521 ± 0.121	0.512 ± 0.145	0.485 ± 0.133
FA	0.501 ± 0.159	0.448 ± 0.112	0.453 ± 0.117

*CTL, control; DKI, diffusion kurtosis imaging; FA, fractional anisotropy; MD, mean diffusivity; MK, mean kurtosis; PD, Parkinson's disease; RN, red nuclei; SN, substantia nigra; SD, standard deviation*.

* *p < 0.05, early-stage PD vs. CTL or advanced-stage PD vs. CT or advanced-stage PD vs. early-stage PD*.

** *p < 0.01, advanced-stage PD vs. CTL*.

### Correlation Between FA, MD, or MK Values in the Substantia Nigra and Unified Parkinson's Disease Rating Scale Scores

There were no significant correlations between MK, FA, or MD values and Unified Parkinson's Disease Rating Scale (UPDRS III) scores.

## Discussion

Early diagnosis of PD using conventional MRI is challenging; more sensitive brain imaging techniques are needed to facilitate early diagnosis and assessment of disease severity. In this study, we used DKI to investigate brain changes in patients with PD. Importantly, these studies revealed that MK values in the bilateral substantia nigra were significantly lower in patients with early- and advanced-stage PD than in controls. Moreover, MK values in the left substantia nigra were significantly lower in patients with advanced-stage PD than in those with early-stage PD. Patients with advanced-stage PD also exhibited significantly decreased MK values in the bilateral red nucleus relative to controls. Hence, DKI may be a useful imaging technique for the diagnosis of PD and assessment of disease severity.

The cardinal motor symptoms of PD include bradykinesia, resting tremor, rigidity, and postural instability, which predominantly result from dysfunction of the substantia nigra pars reticulata and other brain structures (9). The levels of oxidative stress markers in the substantia nigra are greatly increased in patients with PD, which may explain damage to dopaminergic neurons (10). In addition, iron ions, which are abundant in many brain structures, including the substantia nigra and red nucleus (11–13), may play an important role in oxidative stress (14). Thus, pathophysiological changes in the substantia nigra and red nucleus may influence the development and progression of PD.

In our study, we divided patients with PD into early- and advanced-stage PD groups based on UPDRS scores. Our findings indicate that early- and advanced-stage PD were associated with significant decreases in MK values in the bilateral substantia nigra. DKI is a quantitative technique used to reflect non-Gaussian water diffusion in living tissue (5, 17). MK, which is related to structural complexity, is the most commonly used kurtosis index. Thus, as reduced MK values reflect decreases in structural complexity, our findings may be related to neuronal loss and gliosis in the bilateral substantia nigra. Alternatively, iron deposits in the substantia nigra, which aggravate oxidative stress, may also explain the observed decreases in kurtosis. We also observed significant decreases in MK values in the left substantia nigra in patients with advanced-stage PD when compared to those with early-stage PD. These findings indicate that, while the initial onset of motor symptoms occurred on the right side in most patients, the initial lesions occurred in the left substantia nigra. Therefore, our results suggest that MK values can be used to evaluate PD progression.

DKI is a relatively new technique, and previous studies have primarily focused on comparing changes in DKI indices in different brain regions of patients with PD and controls. Few DKI studies have compared these indices among different PD groups. In a study involving 30 PD patients and 30 controls, MK values were increased in the caudate nucleus, putamen, globus pallidus, and substantia nigra in patients with PD (18). More recently, Zhang et al. (19) conducted a large study involving 72 patients with early-stage PD and 72 healthy volunteers. They also observed increased MK values in the substantia nigra of patients with PD relative to those observed in healthy volunteers. In contrast, some studies have reported that MK values are reduced in the white matter of multiple encephalic regions in patients with PD (16, 20). Although, the decreases in MK values observed in the present study differ from those reported in some prior DKI studies, our results may better explain the damage to the substantia nigra in patients with PD. Discrepancies in the levels of MK among these studies may reflect differences in the severity of PD, as MK in the substantia nigra correlates with the severity of motor dysfunction in PD patients (19). For example, Zhang et al. (19) included patients with Hoehn-Yahr stages 1 and 2, while we included patients with Hoehn-Yahr stages 1–4. Differences in methodology may also underlie the lack of agreement between the results of these studies. For example, all image processing operations in a study by Wang et al. (18) were performed using MATLAB 7.8, whereas we utilized Functool software for postprocessing of the DKI data. We speculate that differences in scanning protocols may have contributed to the inconsistencies in MK values between these studies.

MK values in the red nucleus were significantly lower in patients with advanced-stage PD compared to those of patients in the CTL group. However, no such differences were observed between patients with early-stage PD and those in the CTL group or with advanced-stage PD, suggesting that the degeneration of red nucleus neurons arises later during the progression of PD. The degeneration of dopamine neurons is reported to occur not only in the substantia nigra but also in other brain regions ranging from the lower brain stem to the basal ganglia (21). In addition, the red nucleus exhibits a close relationship with the cardinal motor symptoms of PD (22). In accordance with these previous findings, MK values in the red nucleus decreased as symptom severity increased in our patients with PD. This suggests a close relationship between pathophysiological changes in the red nucleus and the cardinal motor symptoms of PD.

As noted in previous studies, we observed no significant differences in FA or MD values among the PD and control groups (23, 24). However, in one previous study, the measurement of diffusion indices in 73 patients with a clinical diagnosis of idiopathic PD and 78 controls using DTI revealed significant decreases in FA values in the substantia nigra in patients with PD (25). Other studies have reported similar results (26–28). Although we observed no changes in FA or MD values, the observed reductions in MK values may indicate that DKI is more sensitive than DTI for the diagnosis of PD.

Consistent with previous findings (18), we observed no correlation between diffusion indices and UPDRS scores. Others, however, have reported a positive association between MK values and UPDRS scores (19). Such discrepancies may be due to the subjectivity of UPDRS scoring and the inability of older PD patients to express their symptoms.

Our study had several limitations. First, although loss of dopamine neurons mainly occurs in the substantia nigra pars compacta, our region of interest included the whole substantia nigra, for which the mean diffusion indices were calculated. In addition, iron deposition may have influenced the measurement of diffusion indices. Future studies should utilize DKI in conjunction with other methods to determine the precise relationship between iron deposition and MK values.

## Conclusion

MK values may aid clinicians in diagnosing PD early, monitoring disease severity, and assessing changes in the bilateral substantia nigra. Changes in the red nucleus are mainly observed in patients with advanced-stage PD. Therefore, MK in the substantia nigra is a potential biomarker of PD in imaging studies and may readily identify patients with Parkinsonism.

## Materials and Methods

### Participants

The present study included 26 patients with PD (mean age, 62.5 ± 8.7 years; Hoehn-Yahr stage, 0–4.0; UPDRS scores, 8–43) and 15 controls (mean age, 59.5 ± 9.4 years). Twenty-four patients with PD exhibited right-sided onset of motor symptoms, while two patients exhibited left-sided onset. Patients with PD were recruited from the Department of Neurology at our hospital, and all CTL participants were recruited from the Medical Examination Center at our hospital. All participants underwent DKI, which was performed using a General Electric (GE) Signa 3.0 T MR Imaging System. PD severity was assessed using the Hoehn-Yahr scale and the UPDRS III. Fifteen patients had a history of medication use, but no adverse reactions were observed among these patients. The PD group was further divided into an advanced-stage PD group (UPDRS ≥ 30; 8 men, 4 women; mean age, 60.1 ± 8.8 years) and an early-stage PD group (UPDRS <30; 5 men, 9 women; mean age, 64.6 ± 8.290 years).

The present study was approved by the Ethics Committee of the Medical College of Shantou University (protocol ID: 2016-05), and all participants provided written informed consent in accordance with the Declaration of Helsinki. Exclusion criteria were as follows: (1) other neurological conditions, including cerebral infarction, cerebral hemorrhage, brain tumor, brain trauma, carbon monoxide poisoning, demyelization, degeneration injury, and vascular dementia; (2) alcohol dependence or a history of taking other psychoactive substances (e.g., antipsychotics or benzodiazepines); (3) history of diabetes or coma due to diabetic ketoacidosis; (4) history of serious medical disease (e.g., heart or respiratory failure, significant liver or kidney dysfunction, anemia, chronic electrolyte disturbance, or heavy metal poisoning); (5) mental illness; and (6) metabolic syndrome lasting longer than 5 years.

### Image Acquisition and Postprocessing

MRI scans were performed using a 3.0-T GE scanner (Signa; General Electric Medical Systems) equipped with a standard eight-channel head coil. We used sponge padding and cotton balls to limit head motion and reduce scanner noise. Initial routine MRI and T2-weighted images [repetition time (TR) = 4,420.0 ms; echo time (TE) = 112.1 ms; 5.0 mm thickness; 1.0 mm septation; matrix 512 × 512; field of view (FOV) = 160 × 160 mm] were obtained for all participants. Two experienced neuroradiologists assisted in the diagnostic process for every view obtained.

**Figure 4:**
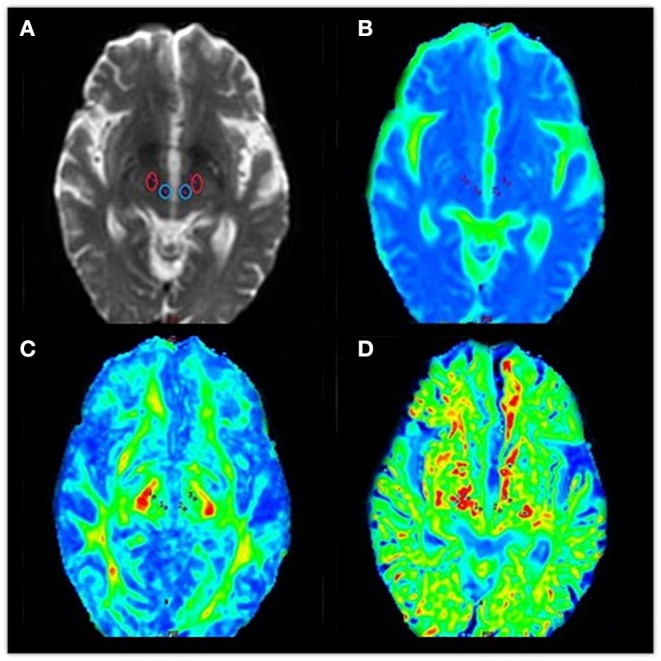
Diffusional kurtosis images of volumes of interest in the bilateral red nucleus and substantia nigra **(A–D)**. **(A)** Volumes of interest in the bilateral red nucleus and substantia nigra. We obtained data from maps of the **(B)** mean diffusivity, **(C)** fractional anisotropy, and **(D)** mean kurtosis.

An echo-planar imaging sequence was used to obtain DKI images with the following scanning parameters: TR, 4,500 ms; TE, 84.1 ms; diffusion gradient pulse duration (δ), 32.2 ms; diffusion gradient separation (Δ), 38.776 ms; FOV, 240 × 240 mm; matrix, 128 × 128; number of excitations, 1; 5.0 mm thickness with no interslice gap; number of slices, 20. Total scan time was 3 min 5 s. Diffusion encoding was applied in 15 directions with three *b* values (0, 1,000, and 2,000 s/mm^2^). All images were postprocessed on commercial workstations (GE, ADW 4.6) equipped with Functool software to generate color-coded and parametric maps of MK, MD, and FA. Volumes of interest in the bilateral substantia nigra and red nucleus were independently drawn three times, with a fixed-in-size (8 mm^2^) ellipse (see Figure 4).

### Statistical Analyses

All data were analyzed using SPSS 20.0 statistical software (SPSS, Inc., Chicago, IL, USA). A one-way analyses of variance test and least significant difference tests were used to evaluate differences in MK, FA, and MD among early-stage PD, advanced-stage PD, and CTL groups. Independent samples *t*-tests were used to compare age and diffusion indices between the PD and CTL groups. Associations between diffusion indices and UPDRS scores were assessed using Spearman's rank correlation coefficient. Chi-square tests were used to evaluate gender differences among the groups. A *p* < 0.05 was considered statistically significant.

## Ethics Statement

The studies involving human participants were reviewed and approved by the Ethics Committee of the Medical College of Shantou University. The patients/participants provided their written informed consent to participate in this study.

## Author Contributions

JG and XM designed and performed the experiments and contributed to the manuscript design and preparation. XM and YG analyzed the results. YG and DQ contributed to the statistical analysis. DQ, EW, YC, ZS, and YS contributed to the editing of the manuscript. JG and RW conceived the research project and helped design the study.

### Conflict of Interest

The authors declare that the research was conducted in the absence of any commercial or financial relationships that could be construed as a potential conflict of interest.

## Abbreviations

CTL: control
DKI: diffusion kurtosis imaging
DTI: diffusion tensor imaging
DWI: diffusion-weight imaging
FA: fractional anisotropy
FOV: field of view
MD: mean diffusivity
MK: mean kurtosis
PD: Parkinson's disease
TE: echo time
TR: repetition time
UPDRS: Unified Parkinson's Disease Rating Scale.
